# Advancing Gene Therapy for Pancreatitis: From Genetic Insights to Clinical Translation

**DOI:** 10.34133/research.1154

**Published:** 2026-02-24

**Authors:** Yi-Zhou Zheng, Yuan-Chen Wang, Hang-Ming Qi, Jie Gao, Zhao-Shen Li, Zhuan Liao, Wen-Bin Zou

**Affiliations:** ^1^ Department of Gastroenterology, Shanghai Institute of Pancreatic Diseases, Changhai Hospital; National Key Laboratory of Immunity and Inflammation, Naval Medical University, Shanghai, 200433, China.; ^2^ Biomaterials for Cancer Therapy and Organ Protection Changhai Clinical Research Unit, Shanghai Key Laboratory of Nautical Medicine and Translation of Drugs and Medical Devices, Changhai Hospital, Naval Medical University, Shanghai, 200433, China.

## Abstract

Pancreatitis is a complex inflammatory disease with substantial genetic determinants. It progresses from acute to chronic forms and poses substantial clinical challenges due to a lack of disease-modifying therapies. Conventional treatments are largely palliative, highlighting the urgent need for mechanism-based interventions. Gene therapy represents a transformative strategy, directly targeting the root genetic causes. This review comprehensively outlines the current landscape and future directions of gene therapy for pancreatitis. We first delineate the genetic underpinnings of the disease, categorizing susceptibility genes into 4 key pathways involving lipid metabolism, trypsin regulation, ductal secretion, and endoplasmic reticulum stress. We then detail the core therapeutic strategies—gene augmentation, suppression, and editing—highlighting both clinically validated drugs (targeting lipid metabolism) and novel preclinical approaches for pancreatitis. Furthermore, to achieve pancreas-targeted delivery, we thoroughly describe the delivery vectors, including viral and nonviral systems, as well as the administration routes. However, translating these therapies faces considerable hurdles, such as physiological and pathological barriers to pancreatic targeting, the challenge of determining intervention timing, the lack of optimal animal models recapitulating human pancreatitis, and host immune responses. We discuss potential solutions to these hurdles, including innovative vector design, improved models, and immunotherapy. Ultimately, gene therapy holds the promise to fundamentally transform the pancreatitis treatment paradigm, offering a path from palliative care to definitive, precision medicine.

## Introduction

The pancreas is a “hidden” organ with both endocrine and exocrine functions. The endocrine component secretes hormones such as insulin to regulate blood glucose homeostasis, while the exocrine component produces enzymes essential for digesting foods. These enzymes are primarily synthesized as inactive precursors (zymogens) by acinar cells and transported to the duodenum via the ductal system, where they are activated by enterokinase as required [1]. Genetic and environmental risk factors promote pancreatitis through the aberrant activation or transport of zymogens.

It has been nearly 4 centuries since the first description of pancreatitis in clinical terms [2]. Pancreatitis manifests as pathological autodigestion of the pancreatic tissue triggered by the obstruction of pancreatic fluid flow and premature zymogen activation. Its 2 major types, acute pancreatitis (AP) and chronic pancreatitis (CP), are both clinically challenging gastrointestinal diseases to treat. AP is an acute inflammatory disease of the exocrine pancreas with tissue damage and necrosis. The global incidence of AP has gradually increased over the past 50 years, reaching an estimated 33.74 cases per 100,000 person-years, with marked geographical differences and the highest rates in the United States [3]. Approximately 20% of AP patients develop severe conditions, including systemic inflammatory response syndrome and multiple organ dysfunction syndrome, with a mortality rate of ~20% [4]. CP is a lifelong progressive fibro-inflammatory disease, with a global prevalence of approximately 50 cases per 100,000 person-years [5]. The disease trajectory exhibits marked clinical heterogeneity, characterized by various complications including pancreatic stones, diabetes mellitus, steatorrhea, and pancreatic cancer [6]. AP-recurrent AP (RAP)-CP are commonly considered a disease continuum, with 21% of AP patients developing RAP, and 38% of RAP progressing to irreversible CP [7].

**Fig. 1.**
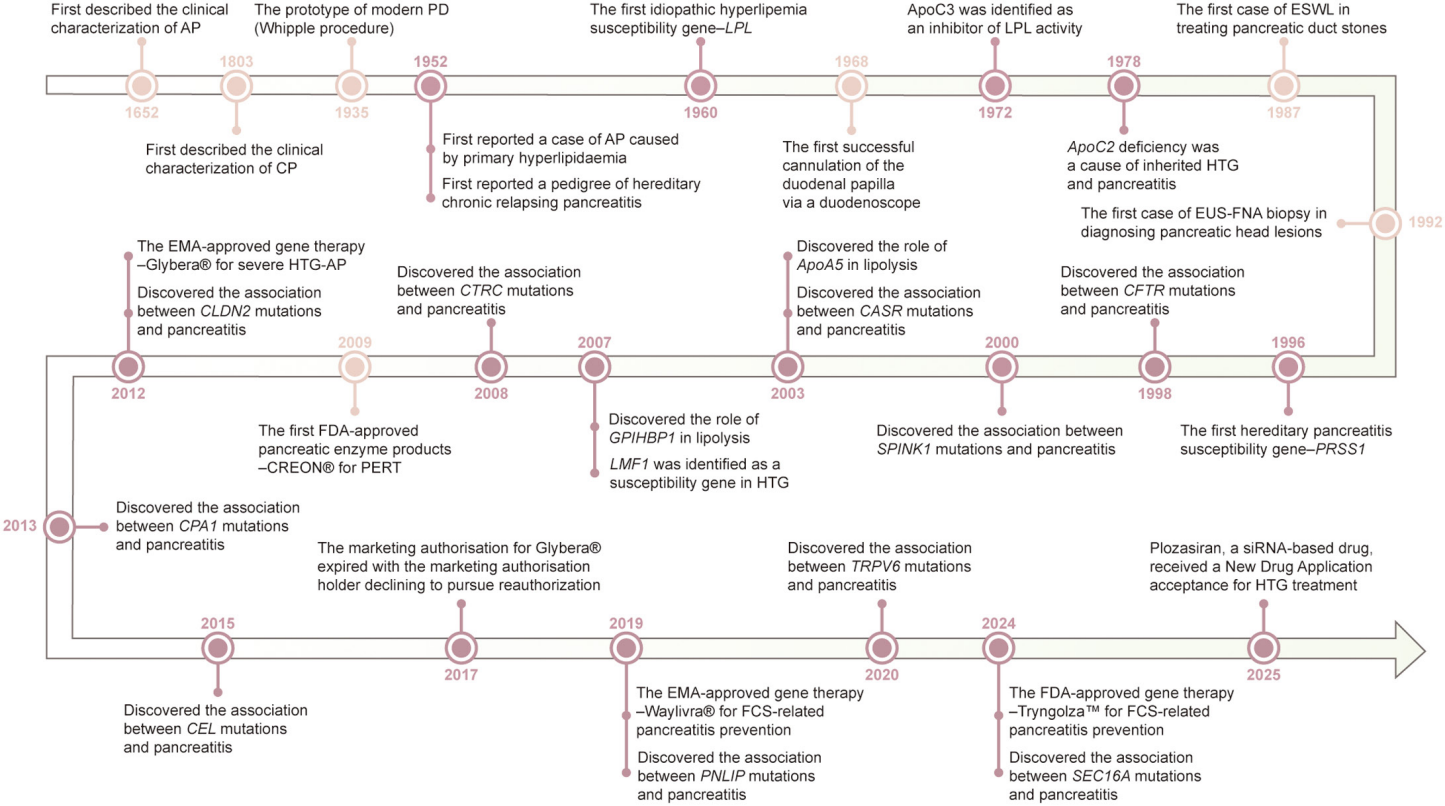
Milestones in the management and genetic research of pancreatitis. This timeline shows the pivotal developments in pancreatitis research from 1652 to 2025. Beginning with the description of disease’s clinical features, the field has witnessed the emergence of various treatments and important discoveries in genetic pathogenesis. The therapeutic modalities have progressed from surgical intervention to minimally invasive therapy, and further advanced into the era of gene-targeted precision therapy. ERCP, endoscopic retrograde cholangiopancreatography; ESWL, extracorporeal shock wave lithotripsy; EUS-FNA, endoscopic ultrasound-guided fine-needle aspiration; FCS, familial chylomicronemia syndrome; HTG, hypertriglyceridemia; PD, pancreaticoduodenectomy; PERT, pancreatic enzyme replacement therapy.

The exploration of pancreatitis treatment has persisted for decades (Fig. 1). From laparotomy-dominated therapy in the early 20th century [8,9] to the emergence of minimally invasive techniques such as endoscopic retrograde cholangiopancreatography (ERCP), extracorporeal shock wave lithotripsy (ESWL), and endoscopic ultrasound-guided fine-needle aspiration (EUS-FNA) in the mid-to-late 20th century, the treatment for pancreatitis has gradually shifted toward conservative and minimally invasive approaches [6]. However, these treatments are primarily symptomatic rather than targeted therapies, leading to a decline in patients’ quality of life and escalating healthcare burdens due to recurrent hospitalizations and complex complication management [4,5].

The pathophysiological complexity of pancreatitis and the absence of a cure underscore the urgent need for mechanism-driven therapies targeting key pathogenic pathways. In 1952, a pedigree of hereditary pancreatitis was first reported [10]. Subsequently, advances in genetics have unveiled disease-associated gene variants across multiple pathways—including lipid metabolism, trypsin regulation, ductal function, and endoplasmic reticulum (ER) stress—as critical pathogenic mediators in pancreatitis progression [11]. It has been shown that almost 33.0% of AP, 45.4% of RAP, and 54.4% of CP cases in pediatric patients harbor pathogenic mutations [12]. Thus, treating the genetic roots of pancreatitis offers a promising path toward a permanent cure.

Gene therapy emerges as a revolutionary treatment modality that directly rectifies genetic defects through gene augmentation, mutation silencing, or precision gene editing [13]. It transcends the limitations of conventional treatments, offering a more definitive solution rather than palliative symptom management. This approach has established novel therapeutic options for patients with hereditary diseases, cancers, and previously incurable diseases, with over 20 gene therapy drugs approved by the U.S. Food and Drug Administration (FDA)/the European Medicines Agency (EMA), and ~4,000 registered clinical trials worldwide [14]. Gene therapy is poised to reshape the treatment of complex diseases, such as pancreatitis, expanding its application far beyond monogenic disorders. Despite advances in pancreatitis gene therapies targeting lipid metabolism and trypsin regulation [15,16], research into other genetically defined pathogenic pathways remains rare (Fig. 1). To advance pancreatitis gene therapy, coordinated efforts are needed to address the following critical challenges, including elucidating pancreatitis-specific genetic pathology, developing safe and efficient pancreatic-tropic delivery systems, and bridging the translational gap from bench to bedside.

In this review, we first summarize the genetic pathogenesis of pancreatitis and rational gene therapy approaches. Second, we synthesize key preclinical and clinical evidence to provide a practical framework for selecting delivery vectors and administration routes in pancreatitis. Last, we analyze the principal translational hurdles and strategies to bridge the gap from preclinical research to clinical practice.

## Genetics and Pathogenesis of Pancreatitis

**Fig. 2.**
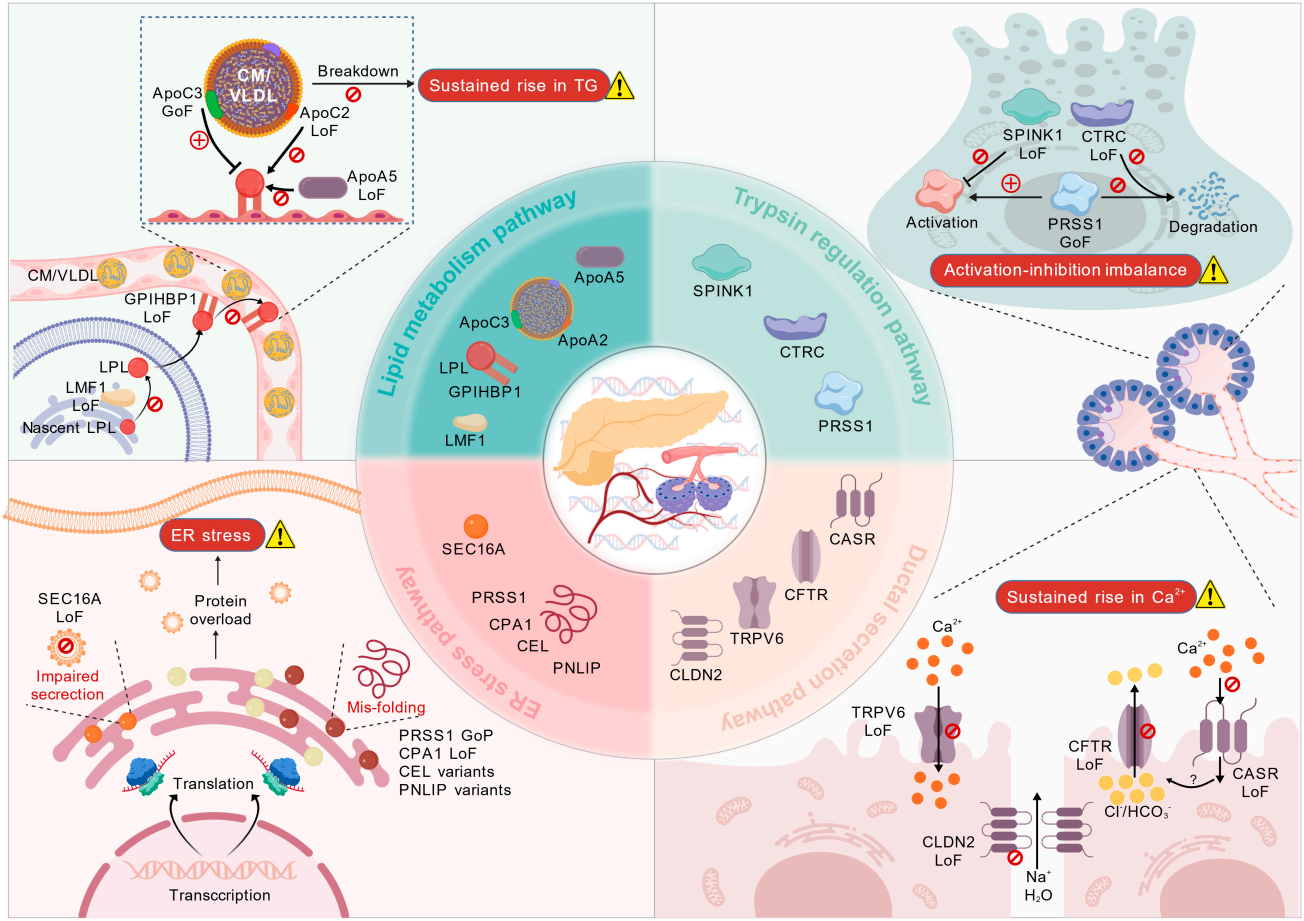
Major genetic pathways associated with pancreatitis. The genetic factors involved in pancreatitis can be categorized into 4 pathways: lipid metabolism, trypsin regulation, ductal secretion, and endoplasmic reticulum (ER) stress pathway.

Advances in genetic research have established the critical importance of genetic factors in the etiology of pancreatitis. The key gene mutations can be categorized into 4 pathways: lipid metabolism, trypsin regulation, ductal secretion, and ER stress (Fig. 2). The lipid metabolism pathway primarily involves loss-of-function (LoF) mutations in lipoprotein lipase (LPL) and its co-activators, elevating serum triglyceride (TG) levels [17]. In the trypsin pathway, gain-of-function (GoF) mutations in serine protease 1 (*PRSS1*), LoF mutations in serine peptidase inhibitor Kazal type 1 (*SPINK1*) and chymotrypsin C (*CTRC*) are the main culprits, leading to trypsin overactivation [18]. The ductal secretion pathway arises from LoF mutations in ion transport-related genes, such as the cystic fibrosis transmembrane conductance regulator (*CFTR*), leading to deficient bicarbonate secretion and elevated Ca^2+^ concentrations, which trigger protein plug formation and premature zymogen activation [19]. The ER stress pathway is mainly related to gain-of-proteotoxicity (GoP) variants in genes encoding acinar secretion proteins, such as certain *PRSS1* variants, and carboxypeptidase A1 (*CPA1*) variants [20]. The distinct events triggered by these 4 major genetic pathways all constitute critical risk for pancreatitis development.

### Lipid metabolism pathway and related genes

Hyperlipidemia, especially hypertriglyceridemia (HTG), is an important etiology of pancreatitis, implicated in up to 30% of AP cases and independently associated with a more severe course of disease [21]. Among primary (genetics-related) hyperlipidemia, types I, IV, and V predominantly manifest the HTG phenotype [22]. It is shown that 46.3% of severe HTG patients had polygenic mutations [23].

Type I dyslipidemia, also known as familial chylomicronemia syndrome (FCS) or lipoprotein lipase deficiency (LPLD), increases severe pancreatitis risk. Specifically, 85% of FCS patients suffer at least one episode of AP and 40% progress to RAP [24]. Some patients further develop CP with a worsening disease course [25]. This autosomal recessive disease is usually caused by mutation of *LPL* (80% to 90%) and other genes encoding proteins required for regulating *LPL* activity, including lipase maturation factor 1 (*LMF1*) (1% to 2%), glycosylphosphatidylinositol-anchored high-density lipoprotein-binding protein 1 (*GPIHBP1*) (5% to 10%), apolipoprotein A5 (*ApoA5*) (2% to 5%), and *ApoC2* (2% to 5%) [26]. *LPL* is responsible for TG breakdown, and its LoF mutations located in exons 4 to 6 often lead to FCS [25]. LMF1, GPIHBP1, ApoA5, and ApoC2 play key roles at different stages of LPL biology and function. LMF1 is an intracellular chaperone protein mediating the proper folding and activation of nascent LPL. Subsequently, GPIHBP1 transports newly secreted LPL across capillary endothelium and stabilizes it on the endothelial surface. At capillary luminal surfaces, LPL interacts with chylomicrons (CMs) and very low-density lipoprotein (VLDL), a process facilitated by ApoA5. Concurrently, ApoC2 acts as the required co-activator of LPL, enhancing its lipolytic function [26]. The LoF mutations in those genes impair LPL-mediated lipolysis, resulting in abnormally elevated TG levels.

Type IV dyslipidemia [familial hypertriglyceridemia (FHTG)] is characterized by an isolated elevation of VLDL. FHTG exhibits familial clustering, and most cases follow a polygenic inheritance model [27]. FHTG-induced AP typically presents in adulthood and requires the combined effects of multiple genetic variants and secondary triggers [28]. Patients with type V dyslipidemia (primary mixed hyperlipidemia/familial combined hyperlipidemia) have a similar AP risk to those with type I dyslipidemia, while the genetic basis of this disorder remains unclear [17].

Specifically, ApoC3 also critically regulates lipid metabolism. It raises TG levels by inhibiting LPL activity and interfering with the function of LDL receptors and LDL receptor-related protein 1 in the hepatic clearance of TG-rich lipoproteins [16]. The GoF variant p.Gln38Lys in *ApoC3* was initially identified in a large kindred of Mexican origin with moderate HTG, resulting in approximately 30% elevated plasma TG levels in heterozygous carriers. Furthermore, LoF mutations in *ApoC3* are associated with improved lipid metabolism [29]. Thus, ApoC3 is a key component in HTG pathogenesis and could be a potential therapeutic target.

### Trypsin regulation pathway and related genes

The imbalance between activation and inhibition of trypsin is a central pathogenic mechanism in the development of pancreatitis. *PRSS1* encodes human cationic trypsinogen, the precursor to trypsin, synthesized and secreted by pancreatic acinar cells. Under physiological conditions, the inactive trypsinogen is transported to the duodenum where enteropeptidase-mediated cleavage converts it to active trypsin for digestion. Pathologically, premature intrapancreatic activation of trypsinogen triggers autodigestive injury through proteolytic enzyme cascades. Pancreatic defense mechanisms against premature activation include *SPINK1*-mediated trypsin inhibition and *CTRC*-dependent trypsinogen degradation [18].

*PRSS1* c.365G>A (p.Arg122His) was the first mutation identified to be associated with hereditary pancreatitis (HP) [30]. Nearly 90% of autosomal dominant HP families carry the *PRSS1* mutation (in the order of frequency: p.Arg122His > p.Asn29Ile > p.Ala16Val ~ p.Arg122Cys > p.Asn29Thr > p.Val39Ala) [18]. Previous studies have provided a comprehensive summary of the critical amino acid residues and systematically classified common *PRSS1* mutations based on their variant types and functional impacts [31,32]. Briefly, the Leu81-Glu82 and Arg122-Val123 peptide bonds are critical sites for *CTRC*-mediated trypsinogen degradation and trypsin autolysis, respectively, while the Lys23-Ile24 and Phe18-Asp19 peptide bonds within the activation peptide are associated with autoactivation. Importantly, mutations in both critical and noncritical sites may act in concert through cross-domain interference, mediated by conformational rearrangements [33]. Two major GoF variants, *PRSS1* p.Arg122His and p.Asn29Ile, have been shown to markedly decrease trypsin autolysis and enhance autoactivation [11]. Moreover, *PRSS1* variants contribute to an increased risk of pancreatic carcinogenesis, with a relative risk as high as 87 [34].

*SPINK1* encodes pancreatic secretory trypsin inhibitor (PSTI), the first line of defense against prematurely activated trypsinogen. Research on *SPINK1* has primarily focused on 2 common LoF mutations: c.101A>G (p.Asn34Ser) and c.194+2T>C. *SPINK1* p.Asn34Ser mutation accounts for 6.4% of idiopathic CP (ICP) patients in European countries [35]. Although this mutation is found to be functionally neutral at the transcription and translation processes by in vitro experiments, its linkage with the enhancer-region variant c.-4141G>T, which down-regulates expression through cis-regulatory mechanisms, forms a risk haplotype [36]. Heterozygous p.Asn34Ser results in a 73% functional loss compared to wild-type in human RNA-sequencing data [37], increasing the risk of RAP [odds ratio (OR) = 19.1] and CP (OR = 11.0) [11,38]. *SPINK1* c.194+2T>C mutation accounts for 43.9% of ICP patients in China [39]. This mutation affects the splicing donor site of intron 3 and leads to exon 3 skipping in *SPINK1* [40], which contains the inhibitor reactive site (Lys18-Ile19 peptide bond) critical for trypsin interaction [41]. The *SPINK1* c.194+2T>C mutation also leads to a substantial decrease in both *SPINK1* mRNA and protein levels, contributing to acinar cell damage and a high risk of CP (homozygous state: OR = 162.4, heterozygous state: OR = 30.39) [39].

*CTRC* functions as the second line of defense against premature trypsinogen activation. Key pathogenic variants, including c.217G>A (p.Ala73Thr), c.703G>A (p.Val235Ile), c.760C>T (p.Arg254Trp), c.738_761del24 (p.Lys247_Arg254del), and c.180C>T (p.Gly60=), increase the CP risk by 5-fold on average mainly involving impaired protein secretion and trypsin-mediated degradation [11,42]. Based on the degree of functional loss (effective activity), p.Ala73Thr and p.Lys247Arg254del are classified as high-risk variants (<10% of wild type), while p.Val235Ile and p.Arg254Trp are classified as moderate-to-low risk (36% to 55% of wild type) [42]. A common haplotype consisting of the synonymous variant c.180C>T has a high detection rate in CP patients (~30%) and increases the CP risk by about 2-fold in the heterozygous state and 10-fold in the homozygous state [43]. However, the functional impact and the disease-relevant variant within this haplotype remain unclear.

### Ductal secretion pathway and related genes

Pancreatic ductal cells produce bicarbonate-rich fluid that plays a critical role in trypsinogen alkalinization, dilution, and transport [44]. Defective genes mainly include *CFTR*, *CLDN2* (claudin 2), *CASR* (calcium-sensing receptor), and *TRPV6* (transient receptor potential channel subfamily V member 6). These gene mutations are involved in the ductal secretion pathway with reduced fluid secretion and disrupted intraductal calcium homeostasis, resulting in a low-flow and high-protein concentration in the pancreas juice, which leads to premature trypsinogen activation and pancreatitis pathogenesis.

CFTR is a chloride (Cl^−^)/HCO3^−^ channel expressed in the apical plasma membrane of secretory epithelial cells in the pancreas, airway, intestine, etc. [45]. *CFTR* mutations were initially identified in association with cystic fibrosis (CF), an autosomal recessive disorder that impacts multiple organ systems [45]. Based on functional impairment, its mutations are categorized into 6 classes, in which classes I to III confer a significant pancreatitis risk by inducing severe channel dysfunction through defects in synthesis, processing, or gating regulation [46]. In general, *CFTR* mutation carriers exhibit an elevated risk of AP (OR = 2.5) and CP (OR = 6.76) [47]. The common mutations *CFTR* p.Phe508del and p.Arg117His increase the risk of CP by 2.5-fold and 4.5-fold, respectively. Moreover, *CFTR* compound and trans-heterozygosity variants represent a stronger risk for CP compared to single variants [48]. *CLDN2*, an X-chromosomal gene, encodes the tight junction protein that functions as water and sodium transporter between ductal cells. Several single-nucleotide polymorphisms (SNPs) at its locus are associated with CP, particularly rs12688220, the effect of which can be further amplified by alcohol consumption [49]. The *CASR* detects extracellular Ca^2+^ concentrations and regulates *CFTR*-mediated ductal fluid secretion through a negative feedback mechanism, thereby balancing the intraductal Ca^2+^ levels. *CASR* mutations can lower its sensitivity to luminal Ca^2+^ and impact ductal fluid secretion. However, the association between CASR variants and pancreatitis risk remains controversial [11]. Collectively, the LoF mutations in *CFTR*, *CLDN2*, and *CASR* could lead to reduced ductal fluid secretion and indirectly affect Ca^2+^ concentration [19].

Lastly, *TRPV6*, a newly identified CP susceptibility gene, encodes a Ca^2+^-selective epithelial ion channel. LoF variants of *TRPV6* have been reported to be associated with CP in several cohorts (OR = 8.9 to 48) [50,51]. Preliminary findings suggest that mutations impair ductal Ca^2+^ clearance and raise intraductal Ca^2+^ levels, with its exact mechanism still under investigation [19].

### ER stress pathway and related genes

The ER stress pathway represents a distinct mechanism of pancreatitis, independent of classical trypsinogen activation and ductal secretion. In this pathway, mutation-induced secretory protein misfolding and impaired secretion trigger ER stress, which culminates in acinar cell damage. Genes related to this pathway mainly include *PRSS1*, *CPA1*, *CEL* (carboxyl ester lipase), *PNLIP* (pancreatic lipase), and *SEC16A* (Sec16 homolog A).

Several *PRSS1* GoP variants, such as p.Arg116Cys, p.Cys139Ser, p.Gly208Ala, and p.Leu104Pro, cause pancreatitis through this mechanism [20]. CPA1 is a digestive enzyme hydrolyzing carboxyl-terminal amino acids from dietary proteins. LoF variants in *CPA1*, including p.Asn256Lys and p.Arg382Trp, are strongly association with CP (OR = 24.9), especially early-onset cases (OR = 84) [20,52]. CEL is responsible for hydrolyzing and absorbing cholesterol esters and lipid-soluble vitamins. The single-nucleotide deletion of *CEL* in the C-terminal variable number of tandem repeats (VNTR) leads to maturity-onset diabetes of the young 8 (MODY8), an autosomal dominant genetic disorder characterized by progressive exocrine pancreatic dysfunction and diabetes [53]. Importantly, a hybrid allele (*CEL-HYB*) is formed through recombination of *CEL* with its adjacent pseudogene *CELP*. The *CEL-HYB1* allele is predominant in European populations and constitutes a significant CP risk factor (OR = 5.2 to 15.5) [54]. Recent evidence suggests that disease risk stratification may be haplotype-dependent, based on specific amino acid residues at positions 488 and 548 [55]. *CEL-HYB1* is rare in Asian populations, while *CEL-HYB2* predominates, with no CP association currently [56]. *PNLIP* is essential for the efficient digestion of dietary fats. Two brothers with *PNLIP* p.Thr221Met homozygous mutation were reported to have CP, while the heterozygous carrier parents remained asymptomatic [57]. Our recent work identified the ER export factors, *SEC16A*, as a novel CP susceptibility gene. LoF variants in *SEC16A* impair ER-to-Golgi transport, leading to an overload of secretory proteins and subsequent ER stress [58].

As outlined above, most pathogenic mutations are LoF variants, while a minority are GoF. These mutations exacerbate pancreatitis risk and progression through distinct mechanisms. Theoretically, corresponding therapeutic strategies targeting these relevant genes, especially gene therapy, could provide a potential cure for pancreatitis, including lowering blood TG levels, re-establishing the trypsin activation–inhibition balance, restoring pancreatic fluid secretion, and correcting protein phenotypes.

## Gene Therapy Strategies for Pancreatitis

**Fig. 3.**
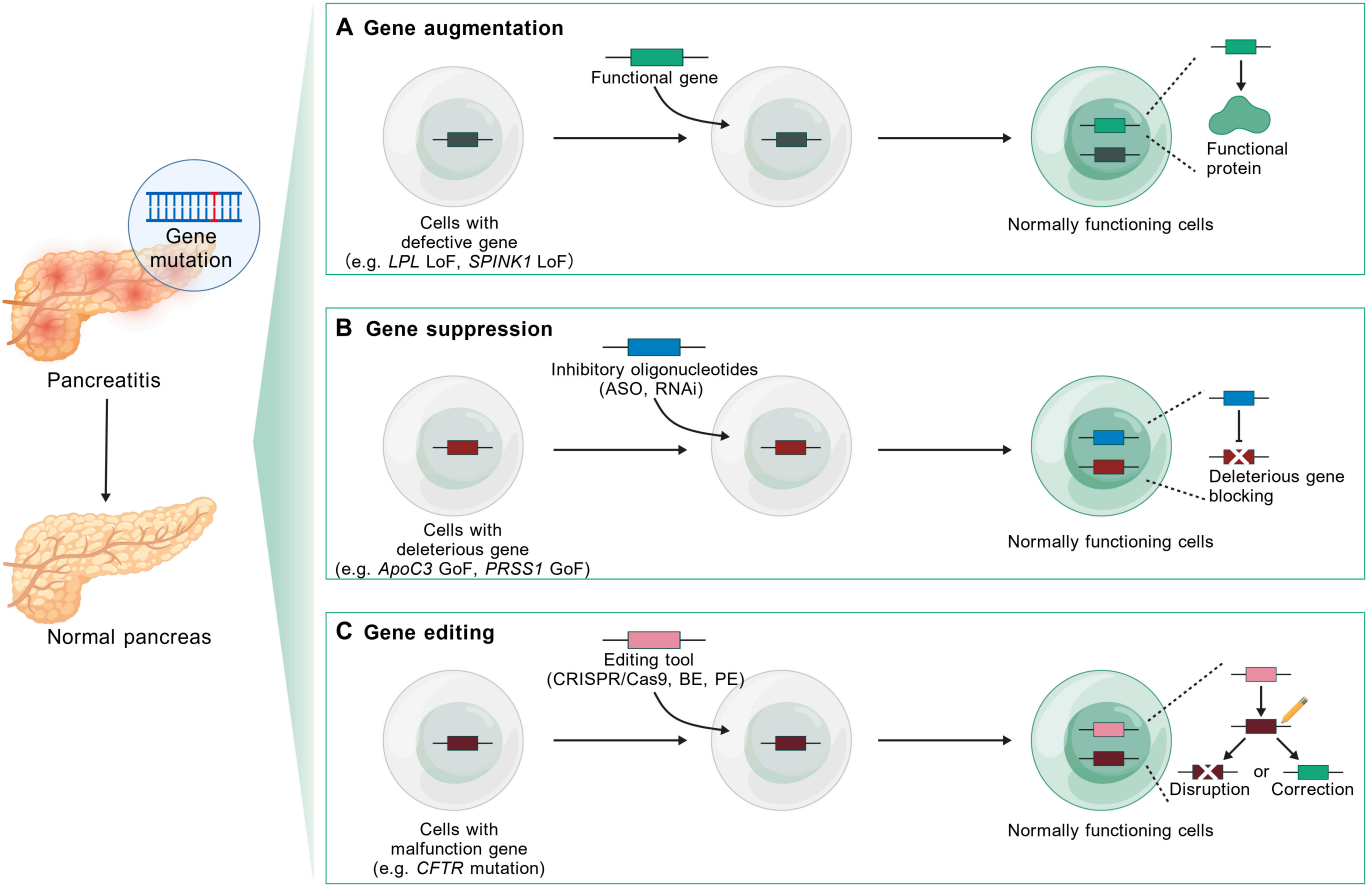
Pancreatitis gene therapy strategies. (A) Gene augmentation refers to delivering functional gene copy into cells to express normal protein, restoring cellular physiological function, which is particularly applicable to pancreatitis caused by LoF variants. (B) Gene suppression utilizes inhibitory molecules like antisense oligonucleotide (ASO) or RNA interference (RNAi) to block the expression of GoF and dominant-negative variant, thereby enabling phenotypic rescue. (C) Gene editing involves the modification of specific target DNA sequences by editing tools [such as CRISPR/Cas9, base editing (BE), or prime editing (PE)], either disrupting the malfunctioning gene to abrogate its harmful effects or directly correcting the causative mutation to restore normal function.

Gene therapy is a therapeutic technique designed to treat or cure diseases with genetic defects. Based on the type of mutation, gene therapy can be categorized into 3 main approaches: gene augmentation (suitable for LoF mutations, e.g., those in *LPL* and *SPINK1*), gene suppression (suitable for GoF and dominant-negative mutations, e.g., those in *ApoC3* and *PRSS1*), and gene editing (theoretically suitable for all types of mutations). In this section, we outline the concepts and characteristics of each strategy (Fig. 3) [13], summarize clinically validated gene therapy agents (Table 1), and highlight promising therapeutic strategies effective in pancreatitis mouse models, thereby establishing the rationale for clinical advancement.

**Table 1.** Completed clinical trials of gene therapy related to pancreatitis

Durg name	Therapeutic agent	Route	Clinical trial identifier	Enrollment	Dosage	Durability	Available outcomes
Alipogene tiparvovec (Glybera)	AAV1- *LPL*^S447X^	IM	CT-AMT-011-01 (NCT 01109498) [167]	14 adult LPLD patients	Cohort 1 (*n* = 2): 3 × 10^11^ gc/kg Cohort 2 ^a^ (*n* = 4): 3 × 10^11^ gc/kg Cohort 3 ^a^ (*n* = 8): 1 × 10^12^ gc/kg	2-year	2 patients showed no improvement in TG levels, while others had a median TG reduction of 39.53%, 3–12 weeks after treatment. TG returned to baseline values in all patients by weeks 16–26. LPL expression and biological activity were demonstrated up to 26 weeks in the injection site. Pancreatitis incidence decreased by 5-fold after 2 years post-vector injection.
			CT-AMT-011-02 (NCT00891306) [60,168]	5 LPLD patients	1 × 10^12^ gc/kg ^b^	14-week	Postprandial total plasma TG and chylomicron TG were reduced by 60% and 85% at week 14, respectively. LPL expression and biological activity were demonstrated up to 52 weeks in the injection site.
			CT-AMT-011-03 [169]	Patients who took part in studies CT-AMT-010-01, CT-AMT-011-01, and CT-AMT-011-02	NA	Retrospective and prospective	Pancreatitis risk decreased by >50% (HR 0.41–0.49) over 3 years post-administration versus pre-treatment baseline.
			CT-AMT-011-05 [61]	Further follow-up of patients who took part in study CT-AMT-011-03 (13 LPLD patients with SAP and RAP)	NA	Retrospective; median 5.8 years post-treatment	A single dose of vector led to a 59% reduction in documented pancreatitis events and a 50% reduction in acute abdominal pain events, with the benefits maintained for up to 6 years.
Volanesorsen (Waylivra)	ASO with 2′-MOE modifications and phosphorothioate substitutions	SC	APPROACH (NCT002211209) [71]	66 FCS patients (33 received treatment)	300 mg (QW for 52 weeks)	52-week	Fasting TG levels were reduced by 77% and 50.1% at weeks 13 and 52, respectively. Patients with a history of recurrent pancreatitis events experienced no attacks during the 52-week treatment period. Self-reported abdominal pain intensity was reduced.
			COMPASS (NCT02300233) [72]	114 HTG patients (75 received treatment)	Cohort 1 (*n* = 25): 300 mg QW for 26 weeks Cohort 2 (*n* = 50): 300 mg QW for 13 weeks and then changed to Q2W	26-week	ApoC3 and TG were reduced by 76.1% and 71.2% after 3 months of treatment. Fasting TG levels were reduced by 78% and 62% in patients with the full dose and the reduced dose at 6 months, respectively. No AP occurred in the treatment group.
Olezarsen (Tryngolza)	GalNAc-conjugated ASO	SC	Balance (NCT04568434) [74]	66 FCS patients (43 received treatment)	Cohort 1 (*n* = 22): 80 mg Q4W for 49 weeks Cohort 2 (*n* = 21): 50 mg Q4W for 49 weeks	53-week	At 6 months, fasting ApoC3 was reduced by 73.7% with 80 mg and 65.5% with 50mg, but the significant reduction of fasting TG (43.5%) was only observed with 80 mg. By 53 weeks, AP events were significantly reduced with olezarsen treated (rate ratio 0.12).
			CORE-TIMI 72a (NCT05079919) [170]	617 HTG patients (409 received treatment)	Cohort 1 (*n* = 205): 50 mg Q4W for 48 weeks Cohort 2 (*n* = 204): 80 mg Q4W for 48 weeks	12-month	At 6 months, TG levels were reduced by 62.9% with 50 mg and 72.2% with 80 mg, and these reductions remained stable through 12 months.
			CORE-TIMI 72b (NCT05552326) [170]	446 HTG patients (296 received treatment)	Cohort 1 (*n* = 149): 50 mg Q4W for 48 weeks Cohort 2 (*n* = 147): 80 mg Q4W for 48 weeks	12-month	At 6 months, TG was reduced by 49.2% with 50 mg and 54.5% with 80 mg, and these reductions remained stable through 12 months. AP events were significantly reduced with Olezarsen treated across CORE-TIMI 72a and CORE-TIMI 72b trials (rate ratio 0.15).
ARO-APOC3 (Plozasiran)	GalNAc- conjugated siRNA	SC	SHASTA-2 (NCT04720534) [76]	226 HTG patients (166 received treatment)	Cohort 1 (*n* = 54): 10 mg at weeks 0 and 12 Cohort 2 (*n* = 55): 25 mg at weeks 0 and 12 Cohort 3 (*n* = 57): 50 mg at weeks 0 and 12	48-week	ApoC3 and TG levels showed a dose-dependent reduction at week 24. 90.6% of treated patients achieved a TG level below the HTG-AP risk threshold at week 24 and 76.5% maintained these reductions at week 48.
			PALISADE (NCT05089084) [77]	75 FCS patients	25 mg (*n* = 26) or 50 mg (*n* = 24) Q3M for 12 months.	12-month	The reductions in ApoC3 and TG levels were comparable at 10 and 12 months for both the 25-mg and 50-mg doses. The incidence of AP decreased with an odds ratio of 0.17 (plozasiran vs. placebo).

gc/kg, genome copies per kilogram; IM, intramuscular injection; QW, quaque week; Q2W, every 2 weeks; Q4W, every 4 weeks; Q3M, every 3 months; SAE, serious adverse events; SC, subcutaneous injection

^a^ Cohorts 2 and 3 received cyclosporine A (3 mg/kg/d) and mycophenolate mofetil (2 g/d) simultaneously with the vector administration, continuing for 12 weeks.

^b^ Cyclosporine A (3 mg/kg/d) and mycophenolate mofetil (2 g/d) were initiated 3 d before vector administration and continued for 12 weeks, and a single dose of methylprednisolone (1 mg/kg) was given 30 min before vector administration.

### Gene augmentation

Gene augmentation is the most straightforward strategy, introducing an exogenous normal gene into target cells to compensate for the endogenous gene function without altering the mutated genomic sequence. This therapeutic strategy is suitable for diseases caused by LoF mutations.

Currently, gene augmentation therapies for AP primarily focus on lipid metabolism-related susceptibility genes. Enhancing the expression of key lipid-lowering genes has been demonstrated to effectively promote TG metabolism and reduce the incidence of HTG-AP. A landmark example is UniQure’s alipogene tiparvovec (Glybera), which delivers the human *LPL* GoF variant S447X (*LPL*^S447X^). This therapy enables sustained synthesis and secretion of functional LPL, achieving a great reduction in plasma TG levels (~40% to 60%) for 12 or 14 weeks post-single injection [59]. The functional copies and biological activity of *LPL*^S447X^ persisted up to 52 weeks, and the frequency and severity of pancreatitis episodes in patients were significantly reduced during a median follow-up of 5.8 years [60,61]. Despite being the first EMA-approved gene therapy for severe HTG-AP patients with LPLD in 2012, the limited durability and high cost (approximately $1.06 million) led to the nonrenewal of its marketing authorization in 2017. A study from China further discovered that overexpression of *LPL* can effectively ameliorate HTG-AP in *Gpihbp1*-deficient animals, providing a potential therapeutic strategy for *GPIHBP1*-related HTG-AP [62].

The treatment of CP is focused on enhancing serine protease inhibition. Previous studies have shown that transgenic expression of rat or human trypsin inhibitor significantly reduced the severity of pancreatitis and trypsin activity in cerulein-induced mice [63,64]. Our team used tyrosine (Y)-to-phenylalanine (F) capsid-optimized adeno-associated virus (AAV) serotype 8 to overexpress human *SPINK1* coding DNA (cDNA), resulting in a 1.4-fold increase in trypsin-inhibitory activity and a 1.6-fold elevation in *SPINK1* protein levels. This approach successfully reduced the severity of pancreatitis and delayed fibrogenesis in 3 preclinical pancreatitis mouse models (cerulein-induced, pancreatic duct ligation, and *Spink1* c.194+2T>C mutant) [15]. Berke and Sahin-Tóth [65] further introduced a short sequence from intron 1 into human *SPINK1* cDNA, boosting mRNA and protein expression over 10-fold in vitro, which successfully addressed the limitation of relatively low *SPINK1* expression levels. Notably, SPINK1 exhibits a similar structure to epidermal growth factor (EGF), suggesting a dual role in pancreatitis protection and potential tumor progression. However, whether SPINK1 drives the progression from pancreatitis to pancreatic cancer remains controversial, and this uncertainty should be considered when conducting SPINK1 overexpression therapy [41,66].

### Gene suppression

Gene suppression utilizes oligonucleotides, such as antisense oligonucleotides (ASOs), small interfering RNAs (siRNAs), and microRNAs (miRNAs), to silence disease-associated genes at the mRNA level. These molecules bind to targeted RNA sequences via Watson–Crick base pairing and disrupt post-transcriptional processes including pre-mRNA splicing, nuclear export, and translation [67], resulting in decreased expression of pathogenic proteins. This strategy is particularly suitable for counteracting the effects of GoF and dominant-negative mutations. To date, ASOs and siRNAs with therapeutic potential have advanced to preclinical and clinical trials for AP.

Unmodified oligonucleotides are highly water-soluble polyanionic macromolecules with limitations such as poor cellular uptake, rapid nuclease degradation, and suboptimal pharmacokinetics [68]. Relevant reviews have summarized modification strategies for oligonucleotides, including chemical modifications and bioconjugations, which can enhance their stability and are crucial for clinical translation [67,69].

#### ASOs

ASOs are chemically synthesized single-stranded oligonucleotides that modulate RNA function through 2 major mechanisms: occupancy-mediated degradation (involving cleavage of target RNAs) or occupancy only (involving steric interference). In the degradation pathway, ASOs bind to the targeted RNA and forms an RNA–DNA heteroduplex, which can be recognized and cleaved by RNase H1, leading to RNA degradation. The occupancy-only mechanism of ASOs includes the disruption of pre-mRNA splicing, inhibition of 5′ capping process, and 3′ polyadenylation, thereby altering mRNA stability and levels [69].

For treating HTG-AP, volanesorsen (Waylivra) is a second-generation ASOs with 2′-O-methoxyethyl (2′-MOE) modifications and phosphorothioate substitutions to selectively target *ApoC3* mRNA (at base position 489 to 508) and inhibit its translation, leading to dose-dependent decreases in plasma ApoC3 and TG levels [70]. In phase III clinical trials (APPROACH and COMPASS), patients with FCS and HTG treated with volanesorsen achieved a significant reduction of ApoC3 and fasting TG levels (~70%) at 3 months. Furthermore, patients with a history of RAP experienced no pancreatitis attacks following volanesorsen treatment, compared with 5 events in 3 placebo-treated patients [71,72]. Based on positive results from multiple clinical trials, volanesorsen received conditional marketing authorization for FCS-related pancreatitis prevention by the EMA in 2019 [70]. Olezarsen, a third-generation GalNAc-conjugated ASO also targets *ApoC3* mRNA, allowing for reduced dosing frequency without compromising efficacy [73]. In a phase III study (BALANCE trial), patients administered monthly 80-mg doses of olezarsen demonstrated a reduction of ApoC3 (>70%) and TG (>40%) levels during a 6-month observation period, with fewer AP events than placebo by 53 weeks [74]. In 2024, olezarsen was approved by the FDA to reduce TG in adults with FCS [73].

#### RNAi

RNAi techniques revolve around 2 critical small noncoding RNA molecules: siRNAs (double-stranded RNAs) and miRNAs (single-stranded RNAs). The silencing mechanisms of these 2 molecules are slightly different. Specifically, siRNAs form near-perfect complementarity with target mRNAs, thereby cleaving specific mRNAs. In contrast, miRNAs generally bind to partially complementary sequences within the 3′ untranslated regions (3′-UTRs) of mRNAs, which may result in broad targets and unpredictable off-target risks [75]. This is a key factor contributing to the slow development of miRNA-based therapeutics. Currently, siRNA-based drugs are being used for the prevention of HTG-AP. Arrowhead Pharmaceuticals has developed the drug ARO-APOC3 (plozasiran), which targets *ApoC3* mRNA to enhance the clearance of VLDL and CM. The phase IIb study (SHASTA-2 trial) showed that 90.6% of plozasiran-treated patients achieved TG levels below the HTG-AP risk threshold (500 mg/dl), effectively preventing AP episodes [76]. The phase III results demonstrated that plozasiran treatment resulted in a 5-fold greater median TG reduction and lower AP incidence versus placebo [77]. These encouraging results have led to plozasiran receiving a New Drug Application acceptance in 2025, showing a promising future.

### Gene editing

The gene editing strategy theoretically offers a one-time therapeutic solution for correcting any disease-causing mutation. Throughout the evolution of gene editing technologies, tools like zinc finger nucleases (ZFNs) and transcription activator-like effector nucleases (TALENs) have gradually been phased out. Given its simplicity, high target specificity, and versatile design, the CRISPR/Cas9 system has been considered the preferred gene-editing platform. The CRISPR/Cas9 system comprises 2 core components: single guide RNA (sgRNA) and Cas9 endonuclease. sgRNA directs the Cas9 to a specific DNA sequence, while Cas9 introduces double-strand breaks (DSBs) at the targeted locus. Subsequently, cellular repair pathways, including nonhomologous end joining or homology-directed repair (HDR), are activated to modify the DNA sequence at the cleavage site [78]. Based on this system, base editing and prime editing [79–81] have been developed, which enable precise single-nucleotide corrections without inducing DSBs.

While gene editing technologies have been extensively explored in pancreatic cancer, their potential for treating pancreatitis remains largely untapped. The gene editing for *CFTR* mutation might offer valuable a blueprint for pancreatitis. Successful correction of the *CFTR* p.Phe508del mutation via HDR-mediated CRISPR/Cas9 editing was demonstrated in cultured intestinal stem cells of CF patients [82]. Furthermore, the fluoPEER system, a prime editing platform coupled with fluorescence-based enrichment, achieved 80% repair efficiency of *CFTR* p.Phe508del in CF patient-derived colon organoids [83]. Among base editors, cytosine base editor (CBE) was developed earlier and has been extensively studied in sickle cell disease, Niemann–Pick disease, etc. [81]. For the critical CP-associated mutation—*SPINK1* c.194+2T>C—CBE theoretically enables the correction of the C-to-T base change, which could serve as an important direction for future research.

CRISPR-based gene editing tools hold great promise for a wide range of applications. However, the potential off-target effects and chromosomal disorganization remain major concerns [84]. As researchers continue refining CRISPR-based gene editing tools [84], parallel attention has focused on emerging RNA editing technologies, which are characterized by reversibility and controllability, thus avoiding permanent genomic alterations and greatly enhancing the safety of gene editing [85]. RNA editing is categorized into single-base editing and RNA exon editing [85]. Single-base editing includes A-to-I, C-to-U, and U-to-Ψ. A-to-I RNA editing is the most extensively studied type and has been successfully applied in the hereditary disorder α-1 antitrypsin deficiency. It enables sequence-specific A-to-I conversion via adenosine deaminase acting on RNA enzymes, which are then recognized as G by the cell translational machinery [86]. U-to-Ψ editing helps the ribosome bypass premature termination codons and serves as an effective approach for nonsense mutations in disease-related genes. This technique rescued ~7% of CFTR expression in cellular models of *CFTR* nonsense mutations [87].

RNA exon editing modifies the splicing process of pre-mRNA by substituting mutant exons with normal ones [88]. This technology ensures generation of intact mRNA transcripts devoid of pathogenic sequences, making it particularly suitable for genetic disorders involving multiple mutations within a single gene. The FDA-approved RNA exon editing therapy ACDN-01 for Stargardt disease demonstrates this strategy’s therapeutic promise [88]. Currently, RNA editing technology is not yet mature, as issues including specificity, on-target editing efficiency, and delivery methods require further resolution [86].

## Delivery Vectors of Gene Therapy for Pancreatitis

**Fig. 4.**
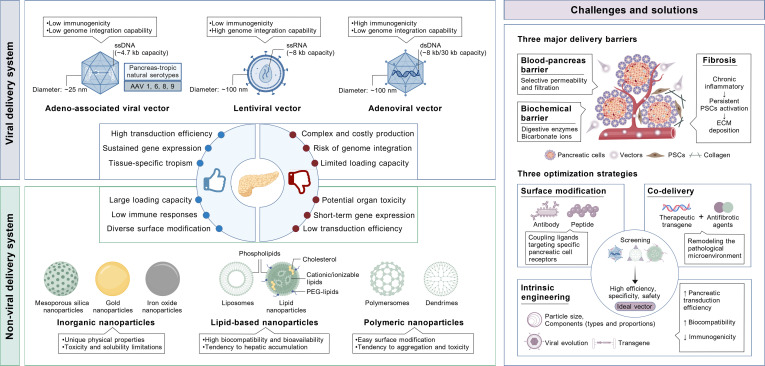
Viral and nonviral vector systems for pancreatitis gene therapy. Viral delivery systems, including adeno-associated viral, lentiviral, and adenoviral vectors, offer tissue-specific tropism and sustained expression but face limitations such as genome integration risks, immunogenicity, or constrained cargo capacity. Nonviral systems, encompassing inorganic, lipid-based, polymeric, and nanoparticles, address immunogenicity concerns but struggle with aggregation and hepatic accumulation. Pancreatic delivery faces multifaceted physiological and pathological obstacles, including blood–pancreas barrier, biochemical barrier, and fibrosis during inflammation. Various strategies, including surface modification, co-delivery, and intrinsic engineering, are used to optimize pancreas-targeted vectors. ECM, extracellular matrix.

Gene therapy demands meticulous selection of delivery vehicles, necessitating comprehensive evaluation of parameters such as immunogenicity, packaging capacity, integration mode, and transduction efficiency (Fig. 4). Delivery vectors can be broadly classified into 2 major categories: viral and nonviral systems. In the context of pancreatitis, it is essential to account for the unique pancreatic inflammatory microenvironment, blood–pancreas barrier (BPB), and fibrosis, which may affect vector biodistribution and penetration [89]. Effective vectors must balance these requirements to ensure successful gene delivery and optimal therapeutic outcomes.

### Viral vector

Viral vectors, including lentivirus, adenovirus, and AAV, are genetically engineered to remove pathogenic components and integrate therapeutic genes, which have been widely used to deliver exogenous genetic material to targeted organs due to strong cellular infection capabilities. As for pancreas, lentiviral vectors achieve high transduction in vitro, but low in vivo due to poor tissue penetration [90,91]. Moreover, a considerable limitation of lentiviral vectors is the random integration of viral genomes, which may cause insertional mutagenesis and disrupt host genome stability, posing a high risk of carcinogenesis [92]. Adenoviral vectors have superior in vivo performance but face constraints from inflammatory responses and transient expression, hindering long-term applications [90]. AAV is a non-enveloped, single-stranded DNA virus with a genome size of ~4.7 kb. Compared to other viral vectors, AAV is considered the most popular viral gene carriers due to its low immunogenicity, specific tissue tropism, stable circulation in the bloodstream, and long-lasting expression of delivered genes [93]. The tissue tropism of AAV is closely determined by serotype-specific capsid–receptor interactions [93]. To date, 13 serotypes and >100 variants of AAV have been characterized, with serotypes 1, 6, 8, and 9 exhibiting marked tropism for the pancreas [91,94–96]. Great efforts have been focused on enhancing the translational potential of AAV-based therapies through capsid optimization and engineering of its cassette—including inverted terminal repeats (ITRs), promoters, and transgene.

#### Capsid optimization

For capsid optimization, rational design is commonly used involving targeted modification of amino acid sequences on existing capsids (e.g., point mutations) or inserting nonviral elements (e.g., cell-targeting peptides) guided by the current structure and biology of AAV capsid to achieve specific function [97]. Surface-exposed Y residues are linked to proteasome-mediated degradation. The Y-to-F capsid-optimized AAV8, developed by mutating 2 surface-exposed Y residues to F at positions 447 and 733, overcomes phosphorylation and subsequent ubiquitination-mediated degradation, and shows higher transduction efficiency and transgene expression in the pancreas [98]. A major difference between AAV serotypes is the affinity of the capsid with host receptors. AAV cellular entry initiates through primary receptors and/or co-receptor interactions, providing a biochemical rationale for engineering capsid-incorporated targeting motifs to redirect tissue specificity. For example, AAV2.5, which was developed by engrafting 5 AAV1 (high muscle tropism)-derived conserved residues, enhanced muscle transfection efficiency up to 5.5-fold compared to AAV2. AAV2.5 has advanced to clinical trials for Duchenne muscular dystrophy, providing direct clinical validation for this strategy [99]. It has been shown that AAVR, GPR108, and TM9SF2 serve as common receptors for 4 pancreatic-tropic AAV serotypes to potentiate cellular internalization and trafficking. Additionally, epidermal growth factor receptor (EGFR) and 37/67 kDa laminin receptor (LamR) also play a role in AAV6, AAV8, and AAV9 infections [93]. However, some other serotypes interact with these receptors but lack pancreas tropism, suggesting the existence of unidentified pancreas-specific receptors mediating AAV tropism. Recently, AAVR2 was identified as an alternative high-affinity receptor for AAV8. A short motif within the AAV8 capsid’s VR-VIII region mediated this interaction, and its transfer conferred AAVR2-dependent transduction to other AAV serotypes. Although the original study focused on hepatic and muscle tissues, genotype–tissue expression data indicate marked AAVR2 expression in human pancreas, suggesting its potential role in pancreatic transduction [100]. Future studies could focus on identifying and characterizing pancreas-specific receptors, as well as systematic analysis of pancreatic-tropic AAV capsids, including conserved amino acid residues, antigenic epitopes, and 3-dimensional structures to rational design of clinically viable vectors. Directed evolution uses mutagenesis techniques, including error-prone PCR, gene shuffling, or random insertion of oligo sequences to generate a large capsid library, which serves as the basis for selecting optimal candidates [97]. In recent years, innovative strategies, such as Cre recombination-based AAV-directed evolution (CREATE), have successfully yielded organ-specific AAV capsid variants [101]. Computer-guided design employs machine learning and computational biology techniques to analyze DNA sequences and phylogenetic datasets across AAV serotypes, thereby predicting and optimizing the performance of AAV capsids [97]. Fit4Function is a novel, open-source machine learning approach used for systematically engineering multi-trait AAV capsids [102]. It enables the quantitative assessment of capsid biodistribution and candidate selection from just one screening round, significantly enhancing the efficiency of AAV capsid development. Besides, Fit4Function performs excellent cross-species translatability across mice, nonhuman primates (NHPs), and human cell models, showing a critical advantage for bridging preclinical-to-clinical translation [102].

#### Cassette optimization

For the cassette optimization, ITRs play a crucial role in viral replication and packaging, whose sequences are located at both ends of the AAV genome. Deleting the terminal resolution site from one side of the ITR can generate self-complementary AAV (scAAV), an AAV with a double-stranded DNA genome. The scAAV can bypass the rate-limiting step of converting single- to double-stranded DNA during cell infection, thereby speeding up gene transcription and transduction. However, scAAV has a lower packaging capacity (~2.3 kb), restricting its applicability in delivering larger transgenes [103].

Furthermore, promoters as another cassette optimization target are important regulatory sequences for the spatiotemporal nature of gene expression. Constitutive promoters (such as *CMV*) are commonly used due to their strong expression, but they suffer from weak specificity and susceptibility to methylation-induced transcriptional silencing [104]. Tissue-specific promoters can direct gene expression in targeted tissues and reduce immune responses against the transgene by minimizing its exposure to antigen-presenting cells [105]. Due to the limited packaging capacity of AAV, it is meaningful to use small, strong, tissue-specific promoters, thereby leaving more available space for therapeutic transgenes. Generally, there are 2 strategies to find tissue-specific promoters. One strategy involves utilizing public databases to identify tissue-enriched genes, and then truncating the upstream sequences from the transcription start site to generate a series of shorter promoter constructs for testing. Another strategy involves the creation of hybrid promoters, which are generated by fusing multiple functional regions from different promoters, such as core promoters, upstream enhancers, and upstream silencers [93]. Lu et al*.* combined the retinal *mGluR6* promoter with additional enhancers, significantly enhancing AAV-mediated transduction specificity and efficiency in mouse rod bipolar cells [106]. Exocrine pancreatic-specific promoters are suitable for AAV-mediated gene delivery but remain unexplored. The aforementioned strategies represent a critical direction for improving the expression specificity of target genes.

Lastly, the optimization of cassette transgene employs intronic sequences, GoF variants, and codon-optimized sequences. It has been demonstrated that some introns can positively regulate gene expression. For *SPINK1*, introducing a 100-base pair short intronic sequence into its cDNA can significantly enhance the efficacy of antitrypsin therapy [65]. Besides, drawing inspiration from the therapeutic transgene used in Glybera, it is valuable to explore GoF variants’ role of other pancreatitis-related genes, to increase protein yield or activity. Codon usage bias (CUB), the preferential use of synonymous codons encoding the same amino acid, is a ubiquitous phenomenon across species and affects translation efficiency, making codon optimization feasible [107]. To quantify CUB, over 30 metrics have been developed, including relative synonymous codon usage, codon adaptation index, transfer RNA (tRNA) adaptation index, and GC content, which are essential to guide gene design [108]. Based on these parameters, optimizing the native gene sequence to match the host-specific CUB without altering the encoded amino acid sequence has proven to be effective in enhancing transgene expression levels and quality in disease models such as Duchenne muscular dystrophy and liver disorders [93]. It should be noted that some tissues exhibit distinct CUBs, suggesting potential benefits from tissue-specific codon optimization. For instance, codon optimization based on a liver-specific CUB has been shown to specifically enhance transgene expression in hepatic cells [109]. To investigate pancreas-specific patterns, an over-representation of CTG and GTG codons was identified in pancreatitis-associated genes [110]. What is more, while investigating xenotransplant rejection, codon-optimized human decay-accelerating factor (DAF) was unexpectedly observed to markedly enhance its expression in mouse pancreatic tissue [111]. These examples reveal the existence of pancreas-specific CUB and highlight codon optimization as a viable strategy for regulating transgene expression.

### Nonviral vector

Despite the widespread use of viral vectors in clinical trials of gene therapy, they are hampered by limitations including restricted packaging capacity, potential toxicity, complex manufacturing processes, and high costs [103]. Consequently, researchers are actively exploring nonviral gene delivery systems based on inorganic compounds, lipids, and polymers. These nonviral vectors provide benefits such as low immunogenicity, high loading capacity, simple synthesis and modification procedures, and great cost-effectiveness, positioning them as promising candidates for gene delivery vectors [112].

#### Inorganic nanoparticles

Inorganic nanoparticles are primarily synthesized from inorganic materials such as gold, silica, and iron [113]. Gold nanoparticles exhibit unique surface plasmon resonance property, making them valuable for bioimaging, photothermal therapy, and controlled release of drugs. Besides, their strong affinity for thiol groups enables stable conjugation with thiol-modified nucleic acids, which not only extends the half-life of the nucleic acids but also enhances their cellular uptake efficiency [114]. Silica nanoparticles, particularly mesoporous silica nanoparticles (MSNs), have the advantages of high porosity, tunable mesoporous structures, and customizable surface chemistry. MSNs with particle sizes of ~100 nm and pore size of 2.5 to 5 nm are suitable for delivering small molecules like siRNA, while larger MSNs (~250 nm particle size, >15 nm pore sizes) provide a high loading capacity for large molecules such as DNA [115]. Surface functionalization through amination or cationic polymer coatings can enhance binding affinity for negatively charged nucleic acids and boost delivery efficiency [116]. Iron oxide nanoparticles (IONPs) possess superparamagnetic properties [117]. The lipidoid-coated IONPs with a size of 50 to 100 nm were reported to display optimal DNA and siRNA delivery activity [118]. Selenium nanoparticles can mitigate the severity of pancreatitis through their inherent anti-inflammatory and antioxidant properties [119]. Notably, clinical translation of inorganic nanoparticles, especially those formulated with heavy metals, remains constrained by low solubility and toxicity concerns [113].

#### Lipid-based nanoparticles

Lipid-based nanoparticles are the most common class of FDA-approved nanomedicines due to their customizable physicochemical properties, high biocompatibility, and bioavailability [113]. It includes 2 main types: liposomes and lipid nanoparticles (LNPs). Liposomes, especially cationic liposomes, are used for nucleic acid delivery. They are vesicles formed by the amphiphilic lipid bilayer, enabling versatile delivery of hydrophilic, hydrophobic, and lipophilic drugs [113]. However, this characteristic also leads to nonspecific interactions with serum albumin and other cellular components, leading to instability, toxicity, and reduced transfection efficiency [120]. Compared to liposomes, LNPs have the micellar structure within the core of the particles, exhibiting more stable structure and higher delivery efficiency [113]. LNPs typically have diameters of 50 to 100 nm and are composed of 4 main components: cationic/ionizable lipids, phospholipids, cholesterol, and polyethylene glycol (PEG)-lipids, contributing to the overall performance through synergistic interactions. Cationic/ionizable lipids enable the encapsulation of nucleic acids via electrostatic interactions, ensuring efficient payload loading. Phospholipids contribute to the structural stability of LNPs and aid endosomal escape, a critical step in intracellular delivery. Cholesterol modulates membrane integrity and rigidity, thereby stabilizing the LNP structure. PEG-lipids have multiple effects on the properties of LNPs, including particle size, circulation half-life, distribution, stability, and targeted delivery ability [121].

One major limitation of LNPs is their tendency to bind with ApoE in the bloodstream, leading to excessive hepatic accumulation, which severely restricts their application for diseases beyond the liver [122]. Studies have indicated that modifying LNP composition can improve nonhepatic delivery. For instance, C-CholF3 LNPs constructed with vitamin D3 achieved a 99% pancreatic targeting efficiency [123]. Besides, 1,2-dioleoyl-sn-glycero-3-phosphocholine (DOPC)-LNPs were found to shift distribution away from the liver and spleen toward the pancreas compared to 1,2-dioleoyl-sn-glycero-3-phosphoethanolamine (DOPE)/1,2-distearoyl-sn-glycero-3-phosphocholine (DSPC) formulations [124]. Furthermore, smaller particle sizes (~30 nm) showed better distribution in the pancreatic exocrine tissue [124]. To achieve targeted delivery to pancreatic tissue, future LNP design must integrate considerations of penetration, circulation, accumulation, internalization, and release [122].

#### Polymeric nanoparticles

The third class of nonviral vectors is polymeric, with advantages of good biocompatibility, structural versatility, and customizability. Polymeric nanoparticles are produced using natural polymers, such as chitosan, dextran, cyclodextrin, and synthetic polymers, such as polyethyleneimine (PEI), poly-l-lysine (PLL), polyamidoamine (PAMAM), and poly (lactic-co-glycolic acid) (PLGA) [117]. Traditional synthetic polymers often exhibit poor biodegradability and require suitable modifications. Strategies such as adjusting the molecular weight or introducing degradable functional groups into the polymer chains have been employed to enhance biodegradation. For instance, incorporating biodegradable polylactide into branched PEI facilitated the safe and effective intracellular siRNA delivery [125]. Furthermore, to enhance their pancreatic targeting specificity, several novel nanoparticles have been developed. For instance, cyclic disulfide-grafted PEI (CD-PEI) has been reported to achieve nearly 100% pancreas-specific transfection with reduced toxicity via intraperitoneal injection [126]. Additionally, neutrophil membrane-coated PEG–PLGA nanoparticles with a 150-nm diameter exhibited significantly enhanced accumulation in inflamed pancreatic tissue [127].

## Delivery Routes of Gene Therapy for Pancreatitis

**Fig. 5.**
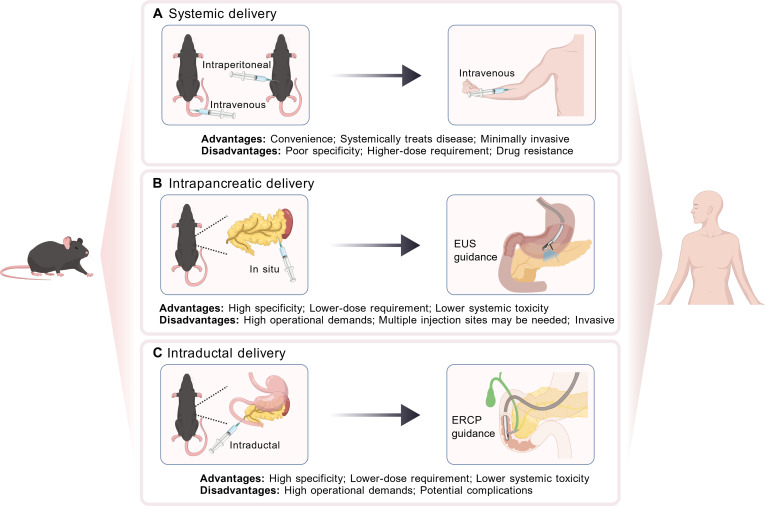
Administration routes for pancreas-targeted gene therapy. Different routes of gene therapy administration and their characteristics. (A) Systemic delivery, via intravenous or intraperitoneal injection, enables body-wide treatment with minimal invasiveness but has poor specificity and requires higher doses. (B) Intrapancreatic delivery directly introduces the therapy into the pancreatic parenchyma, guided by techniques like EUS, providing high specificity and low systemic toxicity but demands experienced skills and multiple injection sites. (C) Intraductal delivery, guided by ERCP, exhibits similar advantages and disadvantages with intrapancreatic administration.

Selecting the appropriate administration route is also critical to achieve specific, durable, and sufficient expression of the therapeutic gene. There are 2 basic strategies for gene delivery: ex vivo and in vivo. Ex vivo approach involves 3 steps: cell isolation, ex vivo transduction, and reinfusion, enabling controlled gene transfer process. It remains limited by technical complexity and is applicable only to specific cell types (e.g., stem cells) [128]. In contrast, in vivo approach allows to introduce therapeutic genes directly into a target organ or tissue of patients or experimental animals to exert intended effects, which simplify the gene therapy process [128]. The in vivo gene delivery strategies can be further categorized into systemic delivery and local delivery (intrapancreatic and intraductal) (Fig. 5).

### Systemic delivery

The systemic delivery (intravenous and intraperitoneal) is closely linked to blood circulation. Intravenous drugs are administered directly into the bloodstream, while intraperitoneal drugs are absorbed through mesenteric vessels and then drain into the portal circulation—a process subject to the hepatic first-pass elimination effect [129]. This blood-borne dissemination pattern contributes to more uniform intrapancreatic gene transfer due to the widespread capillary network, but results in poor tissue specificity [94]. Intravenous injection combined with temporary hepatic triad blockade has been shown to increase vector concentration in the pancreas rather than the liver [94,130]. However, this modified approach faces translational limitations in large animal models due to hepatic ischemia risks. Ayuso et al. [131] applied pancreatic circulation clamping along with adenoviral infusion through the pancreaticoduodenal vein, achieving efficient gene transfer to the canine pancreas without pancreas or liver damage. Notably, during inflammation, alterations in microcirculation and vascular permeability may make it difficult to achieve necessary drug concentration within the pancreatic tissue through systemic delivery [132].

### Local delivery

Compared to systemic delivery, local delivery (in situ injection and pancreatic ductal infusion) offers anatomically targeted administration, allowing for better control of dose, lower cumulative drug dosage, and reduced reliance on drug half-life, which could minimize the risk of adverse events linked to drug toxicity [91,95,133].

Preclinical studies typically use the splenic lobe of the pancreas for in situ injection, which corresponds to the body and tail of the human pancreas [91,96]. During the procedure, the needling depth and angle need to be adjusted to reduce vector leakage and avoid pancreatic injury. Currently, EUS-FNA and interventional therapeutic techniques have frequently been applied in the diagnosis and treatment of pancreatic diseases [6]. In situ injection can potentially be combined with EUS technology for the delivery of vectors.

Retrograde intrapancreatic ductal infusion is also a clinically relevant method, which closely mirrors equivalent routine clinical procedure, known as ERCP. Although pancreatic ductal hypertension is considered as the risk factor of post-ERCP pancreatitis, established pancreatic ductal infusion protocol can be performed without inducing marked pancreatitis in mice [134]. Within the safe range, the infusion time, rate, and concentration of drug can be adjusted based on the type of vectors and the research objectives [134]. Notably, the higher solution viscosity was associated with a greater rise in the pancreatic intraductal pressure, indicating the safety advantage of lower-viscosity formulations [135]. Besides, the vector administered through this method should consider the high concentration of bicarbonate ions and various digestive enzymes present in the pancreatic duct. Otherwise, the biology of the vector may be altered, leading to reduced effect.

## Challenges and Limitations

### Efficiency of pancreas-targeted delivery

The pancreas, as a deep-seated abdominal organ, presents multifaceted physiological barriers and pathological obstacles to effectively delivering drugs (Fig. 4). First, the blood supply to the pancreas is relatively poor and unevenly distributed. Blood flow to the pancreas accounts for only ~1% of cardiac output [136]. The islets, which comprise only 1% to 2% of pancreatic mass, exhibit 5- to 10-fold greater vascular density than the exocrine tissue [136], leading to inadequate drug exposure in exocrine target areas after systemic administration. In addition, the BPB, a natural barrier analogous to the blood–brain barrier, poses a crucial obstacle to drug penetration into pancreatic tissue [89]. The pancreatic microenvironment presents another important consideration for vector delivery. Potent digestive enzymes within the exocrine compartment may actively degrade peptide/protein-based vectors, reducing delivery stability and efficiency. Importantly, it is recommended to evaluate the established procedure in disease animals, as pancreatitis-induced tissue remodeling may alter therapeutic accessibility [130]. In cases of pancreatitis, the microcirculatory disturbances lead to a further reduction in blood flow. It was shown that in acute necrotizing pancreatitis, intravenous antibiotic administration achieves pancreatic tissue concentrations of only ~40% of peripheral blood levels, with rapid subsequent decline [132]. Under chronic inflammatory conditions, pancreatic stellate cells (PSCs) are activated and overproduce extracellular matrix proteins, driving extensive pancreatic fibrosis [5]. This fibrotic tissue forms a physical barrier that further exacerbates the difficulty of vector delivery. Therefore, suppressing PSC activation and developing anti-fibrotic strategies are critical for addressing the pathological obstacles. Nitric oxide (NO), an intracellular signaling molecule, can activate matrix metalloproteinases to promote collagen degradation. The NO donor-loaded liposomes successfully broke fibrotic barriers to treat CP [137]. All-trans retinoic acid-loaded liposomes coated with PDGFRβ-targeting peptide (pPB) and collagenase were also demonstrated to effectively penetrate into fibrotic pancreatic tissue [138]. Besides, a computational-guided in vivo phage display approach identified 5 peptide ligands, allowing peptide-modified liposomes to achieve enhanced and exclusive accumulation in the fibrotic pancreas [139].

### Timing of treatment

The optimal timing of therapeutic intervention in pancreatitis is partially determined by genetics. Pathogenic variants act as molecular accelerators for disease, compressing the timeline from symptom onset to end-stage complications. Compared to AP patients with normal TG levels, those with HTG tend to be younger (mean age, 40 versus 50 years) and have a higher incidence of diabetes [140]. Moreover, in FCS patients with genetic mutations, the average age of the pancreatitis onset is brought forward to the age of 20, greatly accelerating the first manifestation [141]. Pancreatitis caused by *PRSS1* and *SPINK1* variants is characterized by early symptom onset, rapid progression from the first episode of acute AP to CP, accelerated development of exocrine failure and diabetes, as well as high risk of pancreatic cancer [39,142]. It is evident that genetic subtype greatly impacts the trajectory of pancreatitis, making early intervention, rapid onset of action, and sustained therapeutic efficacy critical for the management of patients with high-risk genotypes. While DNA-based gene therapy offers durable expression, its inherently slow onset and delayed peak expression limit its ability to rapidly alleviate acute symptoms. In contrast, mRNA-based gene therapy enables rapid synthesis of functional proteins, providing immediate protection during AP episodes. Although the application of mRNA therapy to modulate pancreatitis-specific genetic pathways remains unexplored, advances such as the LinearDesign algorithm [143]—designed to overcome mRNA instability and low translational efficiency—provide valuable insights into the development of mRNA-based therapies for pancreatitis.

Genetic risk accelerates the pace of disease progression and also affects therapeutic efficacy. In pancreatitis, dynamic pathophysiological changes evolve with disease progression, with notab exocrine pancreatic remodeling. Pancreatic specimens from *SPINK1* [144] and *PRSS1* [145] carriers both showed progressive acinar cell loss, with replacement by parenchymal fibrosis and extensive adipocytes, respectively. It is important to note that the penetrance of different genetic mutations varies. *PRSS1* p.Arg122His and p.Asn29Ile have high CP penetrance (>80%) [34], while in *SPINK1*-related CP, the heterozygous variant c.194+2T>C, which has the highest carrier frequency, has a penetrance of only 4.5% [36]. So, it should be considered whether intervention is necessary before the onset of pancreatitis. In previous gene therapy clinical trials targeting lipid metabolism, participants are generally required to be at least 18 years old and have a clear HTG phenotype along with the corresponding genetic mutation. Unfortunately, to date, CP is mostly diagnosed at an advanced stage when multiple complications exist, and the disease is often irreversible [6]. These stage-dependent histological alterations will impact gene vector transduction efficiency, transgene expression persistence, and the overall therapeutic outcomes, which need to be considered in the choice of treatment timing. A critical challenge in treating CP is the difficulty of achieving a definitive diagnosis in its early stages. To address this unmet clinical need, efforts are being made to discover reliable diagnostic tools. It was reported that immune markers associated with interleukin-17 (IL-17) signaling could distinguish between CP and early-stage pancreatitis [146]. Besides, an 8-metabolite blood signature was verified to distinguish CP from healthy controls, with an area under the curve of 0.85 [147]. In imaging, dual-energy computed tomography (CT) and multiparametric magnetic resonance imaging (MRI) were considered valuable for quantifying pancreatic fibrosis and assessing severity [148]. Artificial intelligence (AI), by integrating clinical scores, laboratory tests, and imaging features, has demonstrated great potential for pancreatitis diagnosis and dynamic prediction, supporting stratified monitoring and early intervention [149,150]. Looking forward, it is speculated that such multimodal biomarker panels and AI-driven models will be crucial for defining the therapeutic window and enhancing clinical decision-making in pancreatitis management by detecting early disease signatures.

### Limitations of preclinical animal models

Preclinical animal models are indispensable for gene therapy development, as they accurately recapitulate human disease and provide a reliable platform for assessing novel treatments. In pancreatitis, genetically engineered mouse models (GEMMs) and cross-species differences highlight the complexity of translating findings from animal models to clinical settings.

#### Genetically engineered mouse models

Researchers have generated various GEMMs to simulate human genetic contributions in pancreatitis (Table 2). Spontaneous pancreatitis is observed in models involving trypsin regulation, ductal secretion, and ER stress pathway. However, many models require exogenous triggers such as cerulein, lipopolysaccharide (LPS), ethanol, or high-fat diet to induce or exacerbate pancreatitis, as exemplified by *TRPV6* models [51]. Multi-gene interactions are evident in *PRSS1*-*PRSS2* [151] and *PRSS1*-*SPINK1* [152] transgenic mice, which lead to spontaneous CP. In the lipid metabolism pathway, none of the GEMMs are reported to develop spontaneous pancreatic injury. Severe disease typically requires a secondary stimulus under conditions of HTG. A notable exception is the *ApoC2* knockout hamster, which exhibits spontaneous pancreatic necrosis and inflammation [153]. Models for *APOA5* and *LMF1* lack reported spontaneous or induced pancreatitis phenotypes in the previous data. Spontaneous pancreatitis models can recapitulate the natural clinical course and are preferred for genetic intervention studies. However, GEMMs of spontaneous pancreatitis are scarce or suboptimal, while most models still rely on exogenous agents to induce disease phenotypes, which may mask the intrinsic effects of genetic mutations and hinder preclinical testing of related targeted therapies. Furthermore, short survival times in certain models limit the window for therapeutic testing.

**Table 2.** Overview of existing genetically engineered mouse models associated with pancreatitis

Gene	Author, year	Species	Genotype (promoter/gene)	Spontaneous pancreatic lesions	Pancreatitis inducer
*PRSS1*	Archer et al., 2006 [171]	Mouse	Rat *Cela1* (−500/+8) promoter/mouse anionic trypsinogen (isoform *T8*) p.Arg122His	7 weeks of age: acinar cell damage; no apoptosis 13–15 weeks of age: interacinar inflammatory infiltrates; 4–7 months of age: 24% fibrotic inflammatory (reaches 40% at more than 1 year of age); duct and islet are grossly normal	Cerulein → severe
	Selig et al., 2006 [172]	Mouse	Rat *Cela1* (−205/+8) promoter/human *PRSS1* p.Arg122His	No	Cerulein → slightly increased severity, maybe due to the low expression of the transgene
	Athwal et al., 2014 [173]	Mouse	Rat *Cela1* (−500/+8) + hGH 3’ UTR (+500/+2,657) promoter/human *PRSS1*, p.Arg122His, p.Asn29Ile	Up to 10% of aging animals showed spontaneous pancreatitis (>9 months) in 3 transgenic strains	Cerulein → severe, but no difference in phenotype was seen among 3 transgenic strains
	Huang et al., 2020 [174]	Mouse	Full-length mouse *Cela1* gene/human *PRSS1* p.Arg122His	No	Both transgenic strains had focal areas of inflammation, but *PRSS1* p.Arg122His showed more severe lesions Cerulein/LPS/ethanol/high-fat diet → *PRSS1* p.Arg122His developed more severe pancreatitis than *PRSS1* or control mice
	Gui et al., 2020 [175]	Mouse	Full-length human *PRSS1* promoter/human *PRSS1* p.Arg122His	No	Cerulein/L-arginine → severe After AP onset, all *PRSS1* p.Arg122His mice developed CP, with histopathological features similar to human HP
	Geisz and Sahin-Tóth, 2018 [176]	Mouse	Mouse cationic trypsinogen (isoform *T7*) p.Asp23Ala knock in	Homozygous state: died around 2 months of age Heterozygous state: AP (~40% at 4–5 weeks of age), CP (2 months of age)	NA
	Jancsó and Sahin-Tóth, 2020 [177] and 2022 [178]	Mouse	Mouse cationic trypsinogen (isoform *T7*) p.Asp24Arg knock in	No	Cerulein → severe After AP onset, mice progressed to CP
	Demcsák et al., 2022 [179]	Mouse	Mouse cationic trypsinogen (isoform *T7*) p.Asp22Asn, Asp24Arg knock in	Homozygous state: spontaneous pancreatitis (6 weeks of age) Heterozygous state: no	Cerulein → severe
	Geisz et al., 2023 [180]	Mouse	Mouse cationic trypsinogen (isoform *T7*) p.Asp23Ala, Lys24Gly or p.Asp22Ala, Lys24Gly knock in	No	Cerulein → severe, but showed no significant difference compared to controls
	Jancsó et al., 2023 [181]	Mouse	Mouse cationic trypsinogen (isoform *T7*) p.Arg123His knock in	No	Cerulein → slightly increased severity, and progressed to CP
*PRSS2*	Wan et al., 2020 [182]	Mouse	Full-length human *PRSS2* promoter/human *PRSS2*	No	Cerulein → severe After AP onset, all *PRSS2* mice developed CP
*PRSS1-PRSS2*	Wang et al., 2022 [151]	Mouse	Full-length human *PRSS1* and *PRSS2* promoter/human *PRSS1*-*PRSS2*, *PRSS1* p.Arg122His-*PRSS2* (crossing 2 transgenic strains with *PRSS2* mice to generate *PRSS2*/*PRSS1*- *PRSS2* and *PRSS2/PRSS1* p.Arg122His-*PRSS2*)	Heterozygous *PRSS1*-*PRSS2* or *PRSS1* p.Arg122His-*PRSS2*: no Homozygous *PRSS1* p.Arg122His-*PRSS2*: AP (3 weeks of age), CP (6–8 weeks of age) Homozygous *PRSS1*-*PRSS2*: no Heterozygous *PRSS2/PRSS1* p.Arg122His-*PRSS2*: spontaneous pancreatitis (3 weeks of age) Heterozygous *PRSS2*/*PRSS1*- *PRSS2*: no	Cerulein (a single low dose, 2.5 μg/kg) → heterozygous *PRSS1* p.Arg122His-*PRSS2* developed AP, but *PRSS1*-*PRSS2* mice did not Cerulein (a single high dose, 100 μg/kg) → heterozygous *PRSS1* p.Arg122His-*PRSS2* and *PRSS1*-*PRSS2* developed similar levels of AP and progressed to CP
*SPINK1*	Ohmuraya et al., 2005 [183]	Mouse	*Spink1*-KO	Homozygous state: autophagic degeneration of acinar cells (16.5 d after coitus), died (14.5 d of age) Heterozygous state: no	Cerulein → no significant change compared to wild-type
	Sun et al., 2020 [184]	Mouse	*Spink1* c.194+2T>C	Homozygous state: died (soon after birth) Heterozygous state: minor acinar loss (11 weeks of age); fibrosis, stellate cell activation and ADM (13 weeks of age)	Cerulein → severe and ADM
	Demcsák et al., 2024 [152]	Mouse	*Spink1*-KO	Homozygous state: died Heterozygous state: no	Cerulein (transient hyperstimulation) → no change compared to wild-type Cerulein (prolonged hyperstimulation) → severe and progressed to CP
*PRSS1*-*SPINK1*	Demcsák et al., 2024 [152]	Mouse	Crossing heterozygous *Spink1*-KO strain with heterozygous *T7* p.Asp23Ala strain or homozygous *T7* p.Asp22Asn, Asp24Arg strain	Heterozygous *Spink1*-KO × heterozygous *T7* p.Asp23Ala: CP (3 weeks of age) Heterozygous *Spink1*-KO × homozygous *T7* p.Asp22Asn, Asp24Arg: CP (4 weeks of age)	NA
*CFTR*	Snouwaert et al., 1992 [185] Ip et al., 1996 [186] Dimagno et al., 2005 [187]	Mouse	*Cftr*-KO	No	Cerulein → severe
	Ratcliff et al., 1993 [188]	Mouse	*Cftr*-KO	~50% animals showed dilatation and blockage of pancreatic duct	NA
	Zeiher et al., 1995 [189] DiMagno et al., 2010 [190]	Mouse	*Cftr* p.Phe508del	No (young adults)	Cerulein → severe
	Delaney et al., 1996 [191]	Mouse	*Cftr* p.Gly551Asp	No	NA
	Tuggle et al., 2014 [192]	Rat	*Cftr*-KO	No (22- to 42-d observation period)	NA
	Rogers et al., 2008 [193] Meyerholz et al., 2010 [194]	Pig	*Cftr*-KO	Exocrine pancreatic insufficiency, duct proliferation	NA
	Ostedgaard et al., 2011 [195]	Pig	*Cftr* p.Phe508del	Reduced parenchyma	NA
	Sun et al., 2010 [196] Olivier et al., 2012 [197]	Ferrets	*Cftr*-KO	Exocrine and endocrine pancreatic insufficiency (1–2 months of age)	NA
	Fan et al., 2018 [198] Van Wettere et al., 2022 [199]	Sheep	*Cftr*-KO	Acinar and ductular dilation by 80 d of gestation ~100% animals showed pancreatic hypoplasia at 120 d of gestation	NA
*TRPV6*	Masamune et al., 2020 [51]	Mouse	*Trpv6* p.Asp541Ala	No	Cerulein → severe
*CPA1*	Hegyi et al., 2019 [200] Orekhova et al., 2020 [201]	Mouse	Human *CPA1* p.Asn256Lys knock in	Homozygous state: progressive CP	Ethanol → most died (2–3 weeks after feeding); the survivors developed CP (3–3.5 weeks after feeding)
*CEL-HYB*	Mao et al., 2022 [202]	Mouse	Human *CEL-HYB1* knock in	~15% at 12 months of age	Cerulein → severe
*PNLIP*	Zhu et al., 2023 [203]	Mouse	*Pnlip* p.Thr221Met	CP (the pathology increased with age and developed slower in heterozygotes)	NA
*SEC16A*	Wang et al., 2024 [58]	Mouse	*Sec16a*-KO	Homozygous state: died Heterozygous state: no	Cerulein → severe
*LPL*	Wang et al., 2009 [204] Weinstock et al., 1995 [205]	Mouse	*LPL* ^*−*/*−*^	NA	Cerulein → severe
*ApoC3*	Zhang et al., 2019 [206]	Mouse	human *ApoC3* transgenic	NA	High TG levels (>2,000 mg/dl) with cerulein → no significant change compared to wild-type
*GPIHBP1*	Zhang et al., 2019 [206]	Mouse	*Gpihbp1* ^*−*/*−*^	NA	High TG levels (>2,000 mg/dl) with cerulein → severe
*ApoA5*	Pennacchio et al., 2001 [207]	Mouse	Human *ApoA5* transgenic; *ApoA5*^*−*/*−*^; *ApoA5*^+/*−*^	NA	NA
*ApoC2*	Gao et al., 2020 [153]	Golden Syrian hamster	*ApoC2* ^*−*/*−*^	Necrosis and inflammation	NA
*LMF1*	Ehrhardt et al., 2014 [208]	Mouse	*LMF1*^*−*/*−*^; *LMF1*^+/*−*^	NA	NA

ADM, acinar to ductal cell metaplasia

Based on experience with GEMMs and recent advancements in understanding the biochemical characteristics of causative genes, both the species and expression level of the transgene are critical for successfully modeling the disease in mice. It is advised to prioritize the construction of humanized GEMMs with high-frequency or severe disease-causing mutations found in CP patients to enhance the clinical relevance and improve phenotype stability. Additionally, developing multi-gene models could better cover the complexity of CP etiologies.

#### Cross-species differences

The translational relevance of murine models is limited by interspecies disparities in pancreatic morphology, physiology, and genetics. The anatomical structure of the mouse pancreas exhibits a greater difference from the human pancreas than that of almost any other laboratory species. Unlike the compact human pancreas, the murine pancreas is diffusely distributed within the mesentery, with pancreatic ducts and accessory ducts converging variably with the bile duct system [154], which may lead to differences in gene transfer in the pancreas via intrapancreatic ductal infusion. In contrast, large animals (e.g., canines and NHPs) exhibit lobular, ductal, and vascular anatomy of pancreas closely resembling human, offering superior anatomical relevance for therapeutic testing [155]. However, large animals entail high costs, long breeding cycles, and a lack of widely accepted pancreatitis models.

To address the shortcomings of cross-species differences, next-generation models, such as patient-derived organoids and human–mouse chimeric models, represent promising alternatives to improve preclinical predictability for pancreatic gene therapy. Pancreatic cancer patient-derived organoids have been utilized for personalized drug testing [156]. However, most pancreatic organoids exhibit limited acinar representation (less than 10%), possibly due to the significant differences between acinar cells and progenitor cells as well as the difficulty in their long-term in vitro maintenance [157,158]. This limitation hinders their utility for pancreatitis drug screening. Recently, Andersson-Rolf et al*.* [159] first demonstrated the existence of tripotent stem/progenitor cells within the human pancreas and established long-term expandable pancreatic organoids with full developmental potential. This system recapitulates all 3 pancreatic cell lineages and may act as a powerful new tool for pancreatitis research and developing therapeutics. Human-mouse chimeric models, through engraftment of human cells or tissues, can overcome species biological differences to better predict pharmacokinetics and vectors’ performance. Pioneering work in liver gene therapy illustrates this potential: Humanized liver chimeras facilitated the clinical translation of AAV-LK03, an engineered vector with enhanced tropism for human hepatocytes [160,161]. Analogous humanized pancreatic model is a promising system, but more basic research is needed to fully understand and realize their potential.

### Immune responses

The clinical translation of gene therapy requires careful consideration of host immune responses, which impact its safety and efficacy. The human immune system constitutes a highly coordinated network encompassing complement activation and innate and adaptive immunity, all capable of targeting both vector components and transgene products. Pancreatitis exhibits a disease-specific immune response characterized by infiltrating immune subset alterations and elevated cytokines [e.g., tumor necrosis factor-α (TNF-α), IL-6, and IL-1β] [162]. Moreover, different types of CP also show distinct T cell receptor repertoires and immune transcriptomic features [146,163]. These characteristics may exacerbate immune responses during gene therapy, limiting its efficacy and safety.

While AAV is favored in clinical trials due to low immunogenicity, emerging evidence reveals considerable immune-mediated side effects in practice, such as hepatotoxicity, particularly following systemic high-dose administration [103]. Approximately 30% to 60% of the global population have preexisting AAV-specific neutralizing antibodies (NAbs) from natural infections [164]. Pre-existing NAbs can reduce AAV efficacy, and newly induced NAbs after initial AAV administration hinder the re-administration, a major issue for chronic conditions requiring repeated dosing. Although serotype switching has been proposed as a solution, it faces limitations in pancreatic applications due to substantial cross-reactivity (>80% sequence homology) among pancreas-tropic natural AAV serotypes [93]. Nonviral vectors also raise similar safety concerns. For instance, in LNPs, the cationic/ionizable lipids can be recognized as danger-associated molecular patterns and the PEG can induce anti-PEG antibodies [121].

Current immunomodulation strategies, including immunosuppression, engineered safety vectors, and techniques to suppress transgene expression in antigen-presenting cells, have been extensively reviewed [97,113], whereas their feasibility in pancreatic gene therapy has rarely been evaluated. It was demonstrated that CD40L suppression and T helper cell depletion can prolong the duration of transgene expression mediated by in situ adenoviral injection and achieve restored expression in previously adenovirus-exposed animal models. However, the minimal restoration level still could not support effective repeated administration in pancreas [165]. Future clinical translation will require systematic evaluation and optimization of immune-compatibility among patient subpopulations, balancing therapeutic benefits against immune risks.

## Conclusions and Prospects

Pancreatitis remains a formidable clinical challenge due to its multifactorial pathogenesis and the absence of disease-modifying therapies. Conventional management strategies predominantly focus on symptom alleviation and complication mitigation, failing to address the underlying genetic etiology. The emergence of gene therapy constitutes a pivotal shift in the therapeutic landscape, enabling direct targeting of pancreatitis-associated gene mutations with potential for curative intervention.

In this review, we clarify the genetic landscape of pancreatitis and describe the applicability of different gene therapy strategies. Due to the complex interplay of genetic factors, a strategic and selective approach is paramount. To guide target selection, we propose that ideal candidates should meet 4 criteria: critical role in disease pathogenesis, selective expression profile, therapeutic accessibility, and minimal immunogenic risk [166]. To date, the most advanced translational milestones have been reached with gene augmentation and gene suppression strategies in reducing lipid levels and preventing pancreatitis recurrence in patients with related genetic deficiencies. Emerging preclinical approaches including lipid metabolism gene intervention, *SPINK1* overexpression, and CRISPR-based editing have shown promise despite their nascent development stage. Besides, we also outlined the pros and cons of various delivery vectors and administration routes, and suggested ways to optimize them for better therapeutic outcomes.

Despite these advances, several critical challenges must be addressed to achieve successful gene therapy. First, the therapeutic window must be precisely defined by disease course, as early treatment may prevent irreversible tissue damage and disease progression. However, determining the optimal timing that balances intervention urgency with overtreatment risks remains a complex issue. Second, the cross-species differences underscore the need for more human-relevant models to improve preclinical predictability. Last, immune responses to delivery vectors and transgene products pose great safety concerns that require innovative mitigation strategies, including vector engineering and immune modulation techniques.

Looking ahead, the field of gene therapy for pancreatitis holds great promise and is faced with both opportunities and challenges. To tackle persistent and pancreatitis-specific challenges, cross-disciplinary lessons in central nervous system disorders and liver disease gene therapy can be drawn. It is believed that with the establishment of a comprehensive technology system spanning from genetic screening to targeted gene therapies, the therapeutic landscape of pancreatitis will shift from reactive and palliative symptom management to proactive prevention and cure for pancreatic injury, ultimately improving outcomes and quality of life for patients suffering from pancreatitis.
